# Pemphigus Foliaceus in a Patient With Pre-existing Atopic Dermatitis: A Case Report

**DOI:** 10.7759/cureus.88268

**Published:** 2025-07-18

**Authors:** Fahad AlSharhan, Reem AlQusaimi, Doaa AlAwadhi, Fawziah AlRujaib, Zainab Alebrahim, Maryam AlHashel, Afnan Alhasawi, Rawan Nasser, Shireefa Alyousef

**Affiliations:** 1 Dermatology, Abdulkareem AlSaeed Dermatology Center, Kuwait City, KWT; 2 Medicine and Surgery, Jaber AlAhmad Hospital, Kuwait City, KWT; 3 Medicine and Surgery, AlAmiri Hospital, Kuwait City, KWT; 4 Medicine and Surgery, AlSabah Hospital, Kuwait City, KWT; 5 Medicine and Surgery, Mubarak AlKabeer Hospital, Kuwait City, KWT

**Keywords:** acantholysis, autoimmune blistering disease, corticosteroids, immunosuppressive agents, pemphigus, pemphigus foliaceus

## Abstract

Pemphigus foliaceus (PF) is a rare autoimmune blistering skin disease characterized by superficial erosions and crusts due to autoantibodies targeting desmoglein 1. Diagnosis can be challenging, particularly when initial presentation mimics common dermatoses such as atopic dermatitis. We report the case of a 55-year-old Syrian female initially diagnosed with severe atopic dermatitis, who presented with widespread pruritic skin lesions unresponsive to corticosteroids. Clinical deterioration with new vesiculobullous lesions prompted re-evaluation. Histopathology and serologic testing confirmed PF. The patient achieved disease control with systemic corticosteroids and immunosuppressive therapy and remains in remission after two years of follow-up. This case underscores the diagnostic complexity of PF, especially in patients with overlapping or misleading dermatologic histories. A high index of suspicion, timely biopsy, and serological confirmation are essential for diagnosis. Long-term immunosuppressive therapy combined with corticosteroids proved effective in achieving sustained remission.

## Introduction

Pemphigus foliaceus (PF) is a rare autoimmune blistering disorder characterized by superficial blistering of the skin due to autoantibodies targeting desmoglein 1 (Dsg1). Unlike pemphigus vulgaris (PV), PF typically spares the mucous membranes and presents with scaly plaques and crusted erosions, most often involving the face, scalp, and upper trunk [1].

Pemphigus, as a group of disorders, has a global incidence estimated at 0.76 to five cases per million individuals annually, with PF being less common than PV in most regions. Onset most commonly occurs between the ages of 50 and 60, with no significant gender predilection [2]. 

We report the case of a middle-aged woman with a long-standing diagnosis of severe atopic dermatitis who developed treatment-resistant, pruritic, vesiculobullous skin lesions. Further investigation revealed PF, emphasizing the importance of revisiting the differential diagnosis in patients with atypical or refractory skin disease.

## Case presentation

A 55-year-old, previously healthy, Syrian female presented to the dermatology clinic with a sudden onset of eczematous lesions with excoriations, predominantly on the trunk. These lesions gradually extended to involve the arms and legs and were intensely pruritic, affecting her quality of life and interfering with her sleep. She is not on any regular medications, has no known drug allergies, and denies any family history of similar dermatologic conditions or autoimmune diseases.

**Figure 1:**
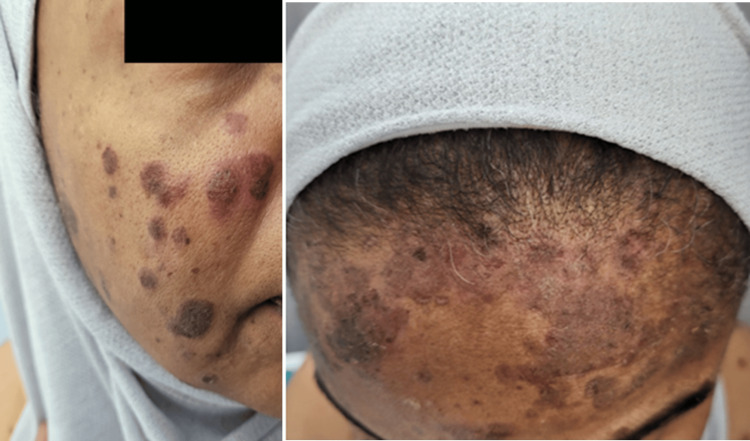
Clinical Findings of Pemphigus Foliaceus Affecting the Face Facial lesions showing multiple erythematous, scaly plaques with superficial erosions and crusting, predominantly affecting the forehead and cheeks.

**Figure 2:**
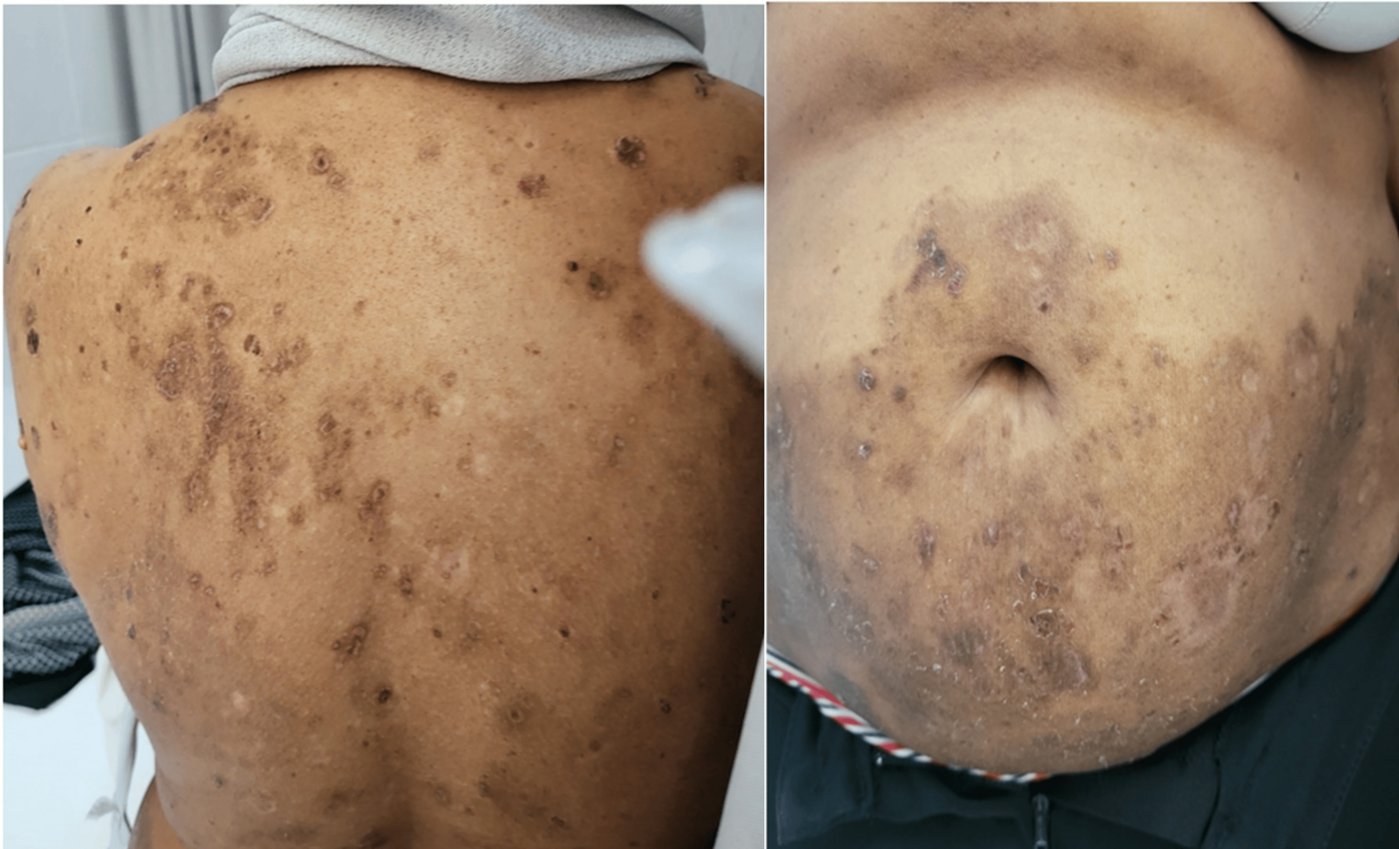
Clinical Findings of Pemphigus Foliaceus Affecting the Trunk Anterior and posterior truncal lesions showing multiple erythematous, scaly plaques with superficial erosions and crusting, along with scattered hypopigmented and hyperpigmented patches, predominantly involving the upper back, shoulders, and abdomen.

Clinical examination revealed multiple violaceous to erythematous, scaly plaques with superficial erosions and overlying serous crusts, primarily distributed over seborrheic areas including the scalp, forehead, cheeks, and extended to the upper and lower trunk, involving both anterior and posterior aspects. The lesions varied in size and appeared to be well-demarcated, with some showing coalescence into larger eroded patches. Several lesions demonstrated active weeping, and surrounding skin exhibited signs of excoriation due to intense pruritus. A positive Nikolsky sign was observed in several areas, indicating intraepidermal acantholysis and epidermal fragility (Figures 1, 2). No mucosal involvement was identified upon examination of the oral cavity, conjunctivae, or genital mucosa. The patient’s vital signs were stable, and no systemic manifestations such as fever, lymphadenopathy, or joint involvement were present at the time of evaluation. The morphology and distribution of the lesions raised suspicion for an alternative diagnosis beyond atopic dermatitis.

Despite adherence to a regimen of high-potency topical corticosteroids, emollients, and oral antihistamines, her condition had progressively deteriorated. A skin biopsy was obtained from a lesion on the abdomen and revealed histopathological features consistent with eczematous dermatitis. The patient was then started on a short course of oral prednisolone 40 mg once daily for 10 days. However, following cessation of the corticosteroid therapy, she experienced a significant relapse with a severe flare-up of her skin lesions. The patient subsequently developed new vesiculobullous lesions predominantly involving the scalp, face, upper trunk, abdomen, and back. These were accompanied by severe pruritus and crusting. In response, she was started on maximal topical therapy, including a high-potency corticosteroid (clobetasol propionate cream) and a topical calcineurin inhibitor (pimecrolimus cream), both applied once daily, along with regular emollient application. Narrowband ultraviolet B (NB-UVB) phototherapy was also initiated to aid in controlling the skin lesions. Laboratory investigations were ordered in preparation for starting dupilumab therapy, given her presumed diagnosis of severe atopic dermatitis. A punch biopsy was also obtained from a lesion on the inner aspect of the thigh for histopathological evaluation.

At the follow-up visit, there was no clinical improvement. The patient continued to develop new vesicular lesions on the arms, thighs, and face, which progressed into bullae predominantly on the inner aspects of both arms and the shins. Routine laboratory screening was performed in anticipation of initiating dupilumab therapy. Results showed that the complete blood count, renal and liver function tests, and lipid profile were all within normal limits. Viral serologies for hepatitis B virus (HBV), hepatitis C virus (HCV), and human immunodeficiency virus (HIV) were non-reactive, and the T-SPOT.TB test was negative. Total serum IgE levels and an allergen-specific IgE panel were performed, both of which yielded negative results, indicating no evidence of atopic sensitization. However, dupilumab was not available at the time. Histopathological examination from the first skin biopsy revealed a subcorneal pustule filled with eosinophils and evidence of acantholysis in the epidermis. The upper dermis showed edema and a mild perivascular lymphocytic infiltrate. These findings were consistent with the diagnosis of PF. The patient was initiated on oral cyclosporine 100 mg three times daily for one month and advised to continue using a potent topical corticosteroid twice daily.

At the third follow-up, the patient’s lesions had been well controlled; however, she had discontinued cyclosporine on her own, which led to the recurrence of new lesions in the previously affected areas. To manage the flare, she was started on oral prednisolone 20 mg daily and mycophenolate mofetil 500 mg twice daily. A second skin biopsy was performed, and further diagnostic workup, including direct (DIF) and indirect immunofluorescence (IIF) studies as well as enzyme-linked immunosorbent assay (ELISA) testing, was initiated.

**Figure 3:**
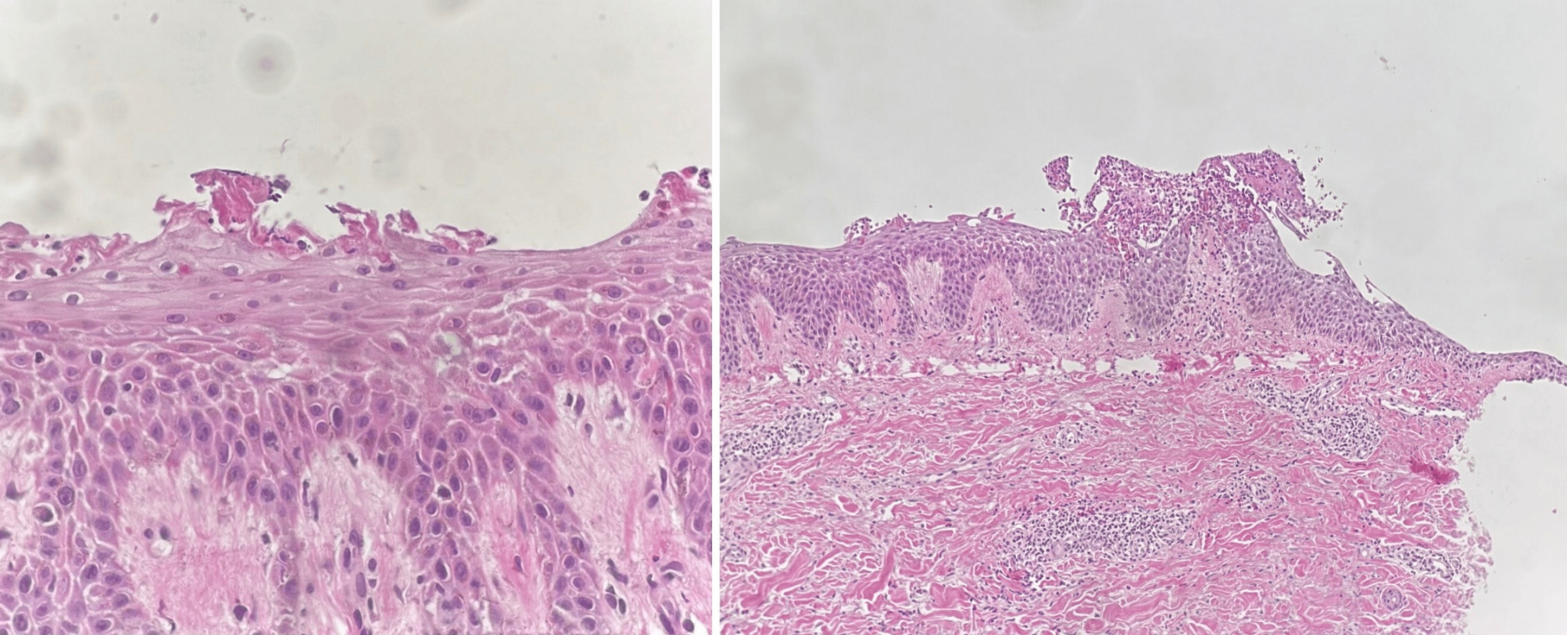
Histopathological Examination of Skin Biopsy Using Hematoxylin and Eosin Stain Histopathological evaluation of the skin biopsy revealed epidermal acanthosis, spongiosis with neutrophilic exocytosis, and an intraepidermal vesicle in the upper epidermis containing neutrophils and acantholytic keratinocytes. The dermis showed a perivascular infiltrate of lymphocytes, neutrophils, and eosinophils.

Ten days after the third visit, the patient showed significant clinical improvement, with complete healing of all body lesions, leaving only post-inflammatory hyperpigmentation. However, new vesicular lesions continued to appear on the face. DIF of perilesional skin revealed intercellular deposition of IgG and C3 in a fishnet-like pattern predominantly within the superficial layers of the epidermis, consistent with a diagnosis of PF. IIF, using monkey esophagus substrate, was negative. ELISA testing revealed a positive Dsg1 antibody and a negative desmoglein 3 antibody, supporting the diagnosis of PF. A second skin biopsy from the right thigh demonstrated epidermal acanthosis, spongiosis with neutrophilic exocytosis, and intraepidermal vesicle formation in the upper epidermis filled with neutrophils and a few acantholytic keratinocytes. The dermis showed a perivascular inflammatory infiltrate composed of lymphocytes, neutrophils, and eosinophils, consistent with PF (Figure 3).

Additional laboratory tests showed an HbA1c of 6.3%, with normal fasting blood glucose, blood pressure, bone mineral density, and complete blood count and biochemical profile. Stool occult blood testing was negative. In light of the persistent facial lesions and overall disease activity, the dose of oral prednisolone was increased to 40 mg once daily. Topical pimecrolimus was initiated twice daily for new lesions, and mycophenolate mofetil was increased to 1 g twice daily. Supportive therapy included a proton pump inhibitor for gastric protection, along with calcium and vitamin D supplementation to prevent steroid-induced osteoporosis. The patient was also advised on adopting a diabetic-friendly diet due to her elevated HbA1c.

At the subsequent visit, the patient's symptoms were well controlled, with no new lesions observed for over two weeks. The treatment plan was continued with mycophenolate mofetil 1 g twice daily, and a gradual tapering of prednisolone was initiated, reducing the dose by 5 mg every two weeks. Over the course of the following year, the patient remained clinically stable on mycophenolate mofetil 1 g twice daily and a maintenance dose of prednisolone 5 mg daily. Routine laboratory monitoring remained within normal limits throughout this period. However, bone mineral density testing revealed osteopenia, prompting a referral to the osteoporosis clinic. As part of long-term management, the dose of mycophenolate mofetil was gradually reduced to 500 mg twice daily, while maintaining prednisolone at 5 mg daily.

**Figure 4:**
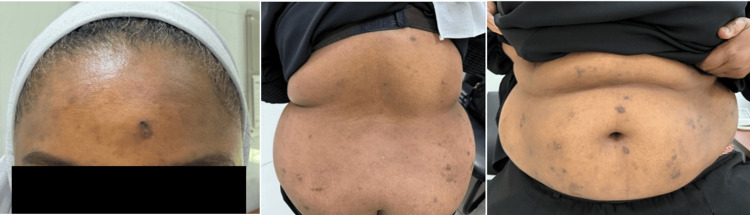
Clinical Follow-up After Two Years of Treatment, Demonstrating Marked Improvement in Skin Lesions The patient exhibits significant resolution of erythema, crusting, and superficial erosions, with re-epithelialization and minimal post-inflammatory changes, indicative of sustained disease control.

At the two-year follow-up, the patient remained clinically stable with no new eruptions reported. She continued on mycophenolate mofetil 500 mg twice daily and prednisolone 5 mg daily. There were no reported side effects or complications related to the ongoing treatment, and the condition remained well controlled (Figure 4).

## Discussion

Pemphigus comprises a group of rare, chronic, autoimmune disorders affecting the mucocutaneous surfaces. It is characterized by the loss of intercellular adhesion, a process known as acantholysis, within the epidermis. This pathological process results in the formation of flaccid bullae and erosions, which may be associated with significant morbidity and, in severe cases, mortality [1]. It has a global incidence estimated at approximately 0.76 to five new cases per million individuals annually. Among the pemphigus variants, PV is generally more prevalent than PF in most regions worldwide [2]. Pemphigus is a globally distributed autoimmune disorder; however, its prevalence demonstrates notable geographic and ethnic variation. The condition is significantly more common among individuals of Ashkenazi Jewish and Mediterranean descent. Despite this increased prevalence in certain populations, pemphigus can affect individuals of all racial and ethnic backgrounds. The disease most commonly presents in individuals between the ages of 50 and 60 years, although the mean age at diagnosis can vary considerably depending on geographic location and ethnic background [1]. Pemphigus disorders generally exhibit no significant gender predilection, affecting both men and women with similar frequency [3]. 

The condition is subclassified into several distinct entities based on clinical presentation and immunopathological findings, including PV, PF, IgA pemphigus, and paraneoplastic pemphigus. Among these, PF has notable subtypes, such as endemic PF, or fogo selvagem, which shares clinical features with the idiopathic form but is associated with environmental or endemic factors, particularly the bite of black flies (Simulium species). Another variant, pemphigus erythematosus (Senear-Usher syndrome), is a localized form of PF that predominantly affects the malar region, producing a rash resembling the "butterfly" rash seen in systemic lupus erythematosus. Moreover, drug-induced pemphigus may manifest as either PF or PV, typically occurring within days to months after the initiation of a causative medication. Thiol-containing compounds, such as penicillamine and captopril, are among the most frequently implicated agents. Overall, drug-induced pemphigus tends to favor the PF subtype over PV, with an observed ratio of approximately 4:1 [4].

PF is a superficial variant of pemphigus, characterized by the presence of autoantibodies directed against Dsg1. Clinically, PF typically presents with cutaneous involvement in the absence of mucosal lesions. This distinguishes it from PV, where extracutaneous symptoms such as ocular discomfort, dysphagia, vocal hoarseness, and dyspareunia are more frequently observed. Lesions in PF commonly appear in seborrheic regions, including the scalp, face, upper trunk, and upper extremities. The cutaneous manifestations are characterized by scattered, superficial blisters that rapidly rupture, evolving into crusted erosions on an erythematous base. These erosions are often described as “bran-like” or resembling “cornflakes” in appearance. As with PV, patients with PF typically exhibit skin fragility and a positive Nikolsky’s sign. Patients frequently report localized pain or a burning sensation at affected sites, though these symptoms are generally milder than those experienced in PV. Furthermore, systemic features such as fever, nausea, or vomiting are uncommon in PF [5]. In its severe form, PF can present as exfoliative erythroderma, characterized by widespread erythema and diffuse scaling across the skin surface. In some cases, this may also be associated with alopecia. Such presentations necessitate urgent hospitalization due to the risk of significant metabolic derangements, which can lead to serious, potentially fatal complications if not promptly managed [3].

The pathogenesis of pemphigus centers on the production of pathogenic immunoglobulin autoantibodies directed against specific keratinocyte surface proteins. Extensive immunochemical and molecular cloning studies have identified the primary antigenic targets in both PV and PF as desmogleins, transmembrane glycoproteins integral to desmosomal junctions. Desmosomes are specialized intercellular structures that provide robust cell-to-cell adhesion, particularly within the stratified squamous epithelium [6]. Specifically, the antigenic target in PF is Dsg1, a cadherin-type cell adhesion molecule within the desmosomal complex. The most pathogenic epitope has been identified in the amino-terminal extracellular domain of Dsg1, as IgG autoantibodies directed against this region can induce intraepidermal blister formation. These autoantibodies disrupt desmosomal integrity by impairing intercellular adhesion among keratinocytes, ultimately resulting in epidermal acantholysis and the formation of superficial, flaccid blisters characteristic of PF without mucosal involvement [4]. 

Several clinical, immunological, and laboratory parameters have been identified as predictors of disease relapse in PF, offering insight into patient prognosis. For instance, patients who exhibit persistent positivity for anti-Dsg1 IgG autoantibodies at treatment completion are at a significantly higher risk of relapse. Additional relapse-associated factors include elevated baseline CD19+ B-cell counts, higher IgG levels, and positive C3 staining in the intercellular spaces on DIF. Several demographic and clinical variables have been evaluated for their potential role in predicting treatment outcomes and relapse risk in pemphigus. Age greater than 65 years has been identified as a positive prognostic factor for achieving complete remission, potentially due to immunosenescence, which may reduce the likelihood of autoimmune reactivation. In contrast, a high body mass index (BMI >35) has been associated with lower rates of complete remission following a single cycle of therapy. Other potential relapse triggers reported in smaller studies include illicit drug use and viral reactivations, such as SARS-CoV-2, herpes simplex virus, and cytomegalovirus, especially in immunocompromised individuals. Additional known precipitants, including thiol-containing drugs, infections, vitamin D deficiency, radiotherapy, pregnancy, trauma, psychological stress, and certain vaccinations, have also been implicated in influencing disease relapse timing [7].

A variety of diagnostic modalities are available to support the clinical suspicion of PF. Histopathological examination and DIF require tissue biopsy specimens. For DIF, it is critical to obtain the biopsy from perilesional skin - clinically uninvolved skin adjacent to an active lesion - as lesional or blistered tissue may be devoid of immune deposits due to epidermal destruction, potentially resulting in false-negative findings. In contrast, IIF and ELISA are serologic tests that utilize patient serum to detect circulating autoantibodies. The diagnosis of PF is established based on a combination of three essential criteria: (1) the clinical presentation, including detailed history and physical examination; (2) histopathological features observed on skin biopsy; and (3) the detection of intercellular autoantibodies via DIF and/or IIF. No single diagnostic method is sufficient in isolation; rather, a comprehensive approach integrating all three components is necessary to confirm the diagnosis [8].

As one of the essential components of the diagnostic triad, histopathological analysis contributes significantly to establishing a definitive diagnosis of PF. The earliest histopathological changes in PF, preceding overt acantholysis, include the development of intercellular vacuolization within the granular layer and/or upper spinous layer of the epidermis. Eosinophilic spongiosis may also be observed during this early phase. As the disease progresses, these vacuoles enlarge, leading to the formation of subcorneal blisters located in the upper epidermis. The blister cavities may contain variable amounts of acantholytic keratinocytes, neutrophils, and fibrin deposits. In more chronic lesions, features of ongoing inflammation and epidermal remodeling are commonly seen. These include papillomatosis, acanthosis, hyperkeratosis, parakeratosis, and follicular plugging. Dyskeratosis of keratinocytes within the granular layer is also evident [4]. Basal melanocytes may exhibit increased pigmentation, and dilated capillaries are frequently noted within the papillary dermis. Additionally, upper dermal edema may be present, along with a mild to moderate perivascular inflammatory infiltrate composed of lymphocytes, eosinophils, and neutrophils in variable proportions [3].

Another essential component in the diagnostic evaluation of PF is immunopathological assessment through DIF and IIF. In PF, DIF reveals a characteristic intercellular space staining pattern, marked by fluorescence outlining the keratinocyte cell membranes. This pattern is typical of pemphigus disorders and reflects the deposition of Dsg1-specific autoantibodies bound to desmosomes on the keratinocyte surface. In PF, the intensity of this staining is often more pronounced in the upper layers of the epidermis, corresponding to the higher expression of Dsg1 in the superficial epidermis and the resulting concentration of antibody deposition. In addition, complement component 3 may also be detected along the intercellular space in some cases of PF [9]. Like DIF, IIF in PF demonstrates an intercellular space staining pattern, often with greater fluorescent intensity observed in the upper epidermis. Immunoglobulin G subclass analysis in PF reveals the presence of both IgG1 and IgG4 antibodies targeting Dsg1, with IgG4 being the predominant subclass involved in pathogenic autoimmunity [5]. IIF titers can serve as a semi-quantitative indicator of disease activity, with higher titers generally correlating with more extensive or severe clinical involvement [3].

The differential diagnosis of PF encompasses a variety of dermatologic conditions that can present with superficial blistering, crusting, and erosions. These include infectious, inflammatory, autoimmune, and drug-induced disorders. One of the most important clinical mimickers is bullous impetigo, particularly in children, as it also presents with fragile, flaccid bullae and honey-colored crusts. Subcorneal pustular dermatosis (Sneddon-Wilkinson disease) can resemble PF histologically due to subcorneal pustule formation, but typically lacks acantholysis and shows a predominance of neutrophils. IgA pemphigus also shares overlapping features but is distinguished by IgA autoantibody deposition and a neutrophil-rich infiltrate. Staphylococcal scalded skin syndrome (SSSS), especially in neonates and immunocompromised adults, presents with widespread superficial epidermal detachment and may mimic PF clinically, although it is caused by bacterial exotoxins and lacks autoimmune features [2]. Other conditions in the differential include seborrheic dermatitis, lupus erythematosus, paraneoplastic pemphigus, drug-induced pemphigus, and pemphigus erythematosus, a localized variant of PF. Distinguishing these entities requires careful correlation of clinical presentation, histopathological features, and immunopathologic findings such as DIF and IIF [10].

The primary goal in the management of PF is to halt disease progression, promote re-epithelialization of affected skin, and prevent secondary infections, all while minimizing treatment-related adverse effects. Systemic glucocorticoids remain the cornerstone of therapy due to their rapid and potent anti-inflammatory and immunosuppressive effects, typically administered at a starting dose of 1-1.5 mg/kg/day of prednisone, with adjustments made based on disease severity and patient response. However, long-term use of systemic corticosteroids is associated with significant adverse events. To improve outcomes and reduce corticosteroid-related toxicity, combination therapy with rituximab has emerged as a preferred approach. Rituximab is typically administered as two doses of 1 g each, given two weeks apart, with subsequent treatment based on clinical response. Co-administration of rituximab allows for lower maintenance doses of corticosteroids, thereby minimizing the risk of serious long-term complications compared to corticosteroid monotherapy [10]. Rituximab is a genetically engineered, chimeric monoclonal antibody targeting the CD20 antigen expressed on B lymphocytes. By depleting pathogenic B cells responsible for autoantibody production, rituximab offers a targeted and durable immunosuppressive effect, making it a highly effective option in the treatment of autoimmune blistering diseases such as PF [11].

Commonly employed alternative adjuvant therapies include mycophenolate mofetil, cyclophosphamide, and azathioprine. Both azathioprine and mycophenolate mofetil are often selected as substitutes for rituximab, particularly in cases where financial constraints limit access or when rituximab is contraindicated due to underlying patient-specific factors. Management of refractory pemphigus remains particularly challenging. In such cases, various adjunctive therapies have been explored, with literature supporting the use of intravenous immunoglobulin (IVIg), immunoadsorption, and plasmapheresis as potential therapeutic options [4]. In localized forms of PF with a limited number of lesions, treatment typically includes moderate- to high-potency topical corticosteroids, intralesional corticosteroids, or topical calcineurin inhibitors. In some cases, dapsone at a dose of 50-100 mg daily may be prescribed as an adjunct to topical therapy, particularly when lesions are persistent or show partial response [9].

Given the chronic, relapsing nature of PF, a multidisciplinary approach to patient management is essential. Preventive strategies should be implemented early to mitigate treatment-related complications. Osteoporosis prophylaxis should be initiated concurrently with the start of systemic corticosteroid therapy due to the risk of bone demineralization. Additionally, histamine-2 receptor antagonists or proton pump inhibitors may be prescribed to reduce the risk of corticosteroid-induced peptic ulcer disease. While patients with pemphigus are often on long-term immunosuppressive therapy, routine prophylaxis for *Pneumocystis jirovecii* pneumonia (PCP) is not universally recommended, although the overall risk of opportunistic infections is elevated. Another important consideration is vaccine safety; patients receiving rituximab or other immunosuppressive agents should avoid live attenuated vaccines due to the risk of disseminated infection [4].

Complications in PF are not uncommon and may result from extensive skin involvement, immunosuppressive treatments, or secondary infections. In widespread disease, disruption of the skin barrier can lead to significant protein and fluid loss, disturbances in electrolyte balance, increased metabolic demand, and heightened susceptibility to both local and systemic infections. These complications not only contribute to increased morbidity but may also delay healing and require additional supportive care [2].

The clinical course of PF is notably heterogeneous, with patients exhibiting variable durations of relapse-free remission interspersed with disease flares that may occur months to several years following initial treatment. Relapse mechanisms are multifactorial and may include incomplete depletion of pathogenic B cells, the emergence of new autoreactive B-cell clones, and the persistence of long-lived CD20+ B cells. The current standard of care for moderate to severe pemphigus involves the use of rituximab in combination with systemic corticosteroids. Despite its effectiveness, relapse rates reported in the literature range from 11% to 40%, depending on the study and treatment protocol. Notably, higher relapse rates and delayed disease control have been linked to suboptimal rituximab dosing regimens, including lower or less frequent administrations. Furthermore, delays in diagnosis, often due to the subtle and evolving nature of clinical presentation, contribute to worsened outcomes and an increased risk of relapse [7].

## Conclusions

This case highlights the diagnostic challenge of PF, particularly in patients with prior misdiagnoses such as severe atopic dermatitis. The development of treatment-resistant, pruritic, vesiculobullous lesions prompted further investigation, leading to the correct diagnosis of PF. Early recognition of PF is crucial to avoid inappropriate treatments and to initiate effective management, which typically involves systemic corticosteroids and immunosuppressive therapy. This case emphasizes the importance of considering PF in the differential diagnosis of refractory skin conditions, particularly when common dermatoses fail to respond to conventional therapies. Further research is needed to explore more targeted therapeutic strategies and to enhance early diagnostic approaches in such atypical presentations.
